# Supplementary material for: Risk management in POCT blood glucose monitoring: FMEA approach aligned with ISO 15189:2022
Source: PLoS One. 2025 Mar 10;20(3):e0319817. doi: 10.1371/journal.pone.0319817 (PMC11892846; doi:10.1371/journal.pone.0319817)
Supplement: S1 File — (PDF) [file pone.0319817.s001.pdf]

Risk Acceptability Matrix Discussion

To refine the definitions and applicability of the Risk Acceptability Matrix, focusing on "Probability of Harm" and "Potential Clinical Impact," and to ensure the matrix accurately reflects the clinical implications of potential risks in POCT blood glucose monitoring.

Topic 1: Probability of Harm

The participants discussed the classification and definitions for 'Probability of Harm,' ensuring the levels are practical and based on expert consensus. The following levels were defined:

Level	Definition	Clinical Example
Frequent	once per week.	Frequent operator non-compliance with SOPs.
Probable	once per month.	Irregular calibration checks or SOP deviations during night shifts
Occasional	once per quarter	Manual data entry errors, such as incorrect patient ID or test results.
Remote	once per year.	Failures caused by rare environmental conditions.
Improbable	once every 5 years or more.	Issues in fully automated processes, such as automated barcode scanning and patient ID matching systems under strict SOP compliance.

Topic 2: Potential Clinical Impact

The participants refined the definitions of 'Potential Clinical Impact' to clearly link failure modes with their clinical consequences in the context of POCT blood glucose monitoring. The following levels were defined:

Level	Definition	Clinical Example
Negligible	No impact on patient safety or clinical decisions.	Non-critical data delay with no diagnostic or treatment impact.
Minor	Minimal impact requiring repeat testing or minor delays, no harm to patient.	Sample contamination requiring recollection without significant delay.
Serious	Moderate impact causing diagnostic delays or inaccuracies, not life-threatening.	Operator errors requiring additional verification.
Critical	Significant impact leading to diagnostic errors or inappropriate treatment, potential patient harm.	Calibration failure causing false hypoglycemia results and overtreatment.
Catastrophic	Severe impact, such as missed critical condition diagnosis, resulting in permanent harm or death.	Missed diagnosis of diabetic ketoacidosis due to critical device failure.

Refined Risk Acceptability Matrix

The refined Risk Acceptability Matrix links the probability of harm and potential clinical impact to categorize risks as acceptable or unacceptable. This systematic approach provides clear guidance for prioritizing risks and implementing control measures.

Probability of Harm	Negligible	Minor	Serious	Critical	Catastrophic
Frequent	unacceptable	unacceptable	unacceptable	unacceptable	unacceptable
Probable	acceptable	unacceptable	unacceptable	unacceptable	unacceptable
Occasional	acceptable	acceptable	acceptable	unacceptable	unacceptable
Remote	acceptable	acceptable	acceptable	unacceptable	unacceptable
Improbable	acceptable	acceptable	acceptable	acceptable	acceptable

Conclusion

The refined Risk Acceptability Matrix enhances the clarity and applicability of risk evaluation in POCT blood glucose monitoring.
