# Supplementary material for: Risk management in POCT blood glucose monitoring: FMEA approach aligned with ISO 15189:2022
Source: PLoS One. 2025 Mar 10;20(3):e0319817. doi: 10.1371/journal.pone.0319817 (PMC11892846; doi:10.1371/journal.pone.0319817)
Supplement: S2 File — (PDF) [file pone.0319817.s002.pdf]

Complete Risk Assessment Report: Pre-Analytical, Analytical, and Post-Analytical Phases

Pre-Analytical Phase Risk Assessment Report

1. Management - Unclear Management Framework and Undefined Responsibilities

Issue Description:

An unclear management framework and undefined responsibilities may hinder effective implementation of quality control, equipment maintenance, and process management.

Hospital Situation:

- **Peking University Shenzhen Hospital**: The existing management framework is relatively complete but lacks regular updates and clearly defined responsibilities.
- **Wuhan Third Hospital**: A formal management framework has not yet been established, with some responsibilities relying on individual experience.

Potential Consequences:

Undefined management responsibilities could result in disorganized process implementation, increasing the risk of overlooked issues.

Scoring Analysis:

- **Severity Score (S)**: Serious (4)
  Reason: Management deficiencies may cause deviations in process execution but are unlikely to directly lead to critical incidents.

Probability Score (P):

Peking University Shenzhen Hospital: Remote (2)

Reason: There is an existing management system, and issues of responsibility allocation occur occasionally.

Wuhan Third Hospital: Remote (2)

Reason: Although management depends on individual experience, no major problems have been caused so far.

Risk Level:

Peking University Shenzhen Hospital: Acceptable (Remote, Serious)

Wuhan Third Hospital: Acceptable (Remote, Serious)

Control Measures:

No further action plan is required at this time.

2. Equipment Evaluation and Selection - Inadequate Performance Validation Prior to Hospital Entry

Issue Description: The lack of sufficient performance validation for equipment prior to clinical use can result in biased results or failure to meet clinical requirements.

Hospital Situation:

Peking University Shenzhen Hospital: The equipment introduction process does not strictly enforce performance validation, and critical parameters, such as hematocrit levels and measurement range, are not adequately assessed.

Wuhan Third Hospital: The performance validation process is relatively standardized; however, the validation records are incomplete.

Potential Consequences:

Failure to validate equipment performance may result in inaccurate test results, which could adversely impact diagnosis and treatment decisions.

Scoring Analysis:

Severity Score (S): Critical (4)

Reason: A lack of performance validation directly compromises test quality, leading to potential misdiagnosis or delayed treatment.

Probability Score (P):

Peking University Shenzhen Hospital: Occasional (3)

Reason: Some equipment is introduced without undergoing a formal validation process.

Wuhan Third Hospital: Improbable (1)

Reason: The validation process is relatively standardized, significantly reducing the likelihood of errors.

Risk Level:

Peking University Shenzhen Hospital: Unacceptable (Occasional, Critical)

Wuhan Third Hospital: Acceptable (Improbable, Critical)

Next Steps:

Establish Rigorous Equipment Performance Validation Procedures:

Objective: Ensure all equipment undergoes standardized performance validation prior to clinical implementation.

Details: Evaluate and document critical parameters, including hematocrit levels and measurement range, during the validation process.

Conduct Regular Cross-Validation:

Action: Periodically compare equipment results with standard laboratory analyzers to ensure consistency, reliability, and compliance with clinical standards.

3. Multiple Instrument Brands - Challenges Arising from the Use of Various Brands

Issue Description: The use of multiple instrument brands introduces inconsistencies in operations and results, increasing complexity in both management and usage.

Hospital Situation:

Peking University Shenzhen Hospital: The use of multiple brands results in variations in operating procedures and result interpretation.

Wuhan Third Hospital: Although fewer brands are used, some instruments still exhibit consistency issues.

Potential Consequences:

Inconsistencies in operation and results may lead to test deviations, thereby increasing the risk of misdiagnosis.

Scoring Analysis:

Severity Score (S): Serious (3)

Reason: Brand differences may cause operational and interpretative errors but are unlikely to directly endanger life.

Probability Score (P):

Peking University Shenzhen Hospital: Occasional (3)

Reason: The variability increases with the use of multiple brands.

Wuhan Third Hospital: Occasional (3)

Reason: Although fewer brands are used, potential issues remain.

Risk Level:

Peking University Shenzhen Hospital: Acceptable (Occasional, Serious)

Wuhan Third Hospital: Acceptable (Occasional, Serious)

Control Measures:

No further action plan is required at this time.

4. Environmental - Non-Compliance with Environmental Conditions

Issue Description: Non-compliance with environmental conditions (e.g., temperature, humidity) may negatively impact the performance of equipment and reagents.

Hospital Situation:

Peking University Shenzhen Hospital: Environmental monitoring at nursing stations is insufficient, and some areas fail to meet temperature and humidity requirements.

Wuhan Third Hospital: Humidity levels in certain storage areas are elevated, affecting the performance of reagents and equipment.

Potential Consequences:

Improper environmental conditions may lead to equipment performance fluctuations and test result deviations.

Scoring Analysis:

Severity Score (S): Serious (3)

Reason: Environmental non-compliance can affect test quality but does not typically pose a direct life-threatening risk.

Probability Score (P):

Peking University Shenzhen Hospital: Remote (2)

Reason: Most areas meet environmental requirements.

Wuhan Third Hospital: Remote (2)

Reason: Issues in high-risk areas are relatively controlled.

Risk Level:

Peking University Shenzhen Hospital: Acceptable (Remote, Serious)

Wuhan Third Hospital: Acceptable (Remote, Serious)

Control Measures:

No further action plan is required at this time.

Analytical Phase Risk Assessment Report

1. Document and Records of SOP - Lack of Standardized SOPs and Absence of Appropriate Regulations

Issue Description: The absence of standardized operating procedures (SOPs) results in inconsistent processes and regulations, increasing the likelihood of errors.

Hospital Situation:

- **Peking University Shenzhen Hospital**: Some processes have SOPs, but they are infrequently updated and do not comprehensively cover all workflows.
- **Wuhan Third Hospital**: Most processes lack formal SOPs and rely on experience-based operations.

Scoring Analysis:

- **Severity Score (S)**: Minor (2)
  - *Reason*: Non-standardized processes increase the likelihood of errors but do not directly harm patients.
- **Probability Score (P)**:
  - *Peking University Shenzhen Hospital*: Occasional (3)
  - *Wuhan Third Hospital*: Occasional (3)

Risk Level:

Peking University Shenzhen Hospital: Acceptable (Occasional, Minor)

Wuhan Third Hospital: Acceptable (Occasional, Minor)

Control Measures:

No further action plan is required at this time.

2. Personnel Training - Insufficient Personnel Training

Issue Description: Inadequate training leads to non-standardized operations, inconsistent test results, and insufficient ability to handle equipment or abnormal results.

Hospital Situation:

- **Peking University Shenzhen Hospital**: Some operators have not completed comprehensive training, especially for new equipment.
- **Wuhan Third Hospital**: Training lacks systematic structure and mainly relies on on-the-job guidance, with insufficient supervision for trainees.

Scoring Analysis:

- **Severity Score (S)**: Serious (3)
  - *Reason*: Improper operations can result in equipment misuse and test inaccuracies, particularly in critical value testing, which may pose safety risks.
- **Probability Score (P)**:
  - *Peking University Shenzhen Hospital*: Probable (4)

Reason: Training coverage for new equipment is insufficient, leading to a higher error probability.

Wuhan Third Hospital: Probable (4)

Reason: Training relies on experience with insufficient systematic structure.

Risk Level:

Peking University Shenzhen Hospital: Unacceptable (Probable, Serious)

Wuhan Third Hospital: Unacceptable (Probable, Serious)

Next Steps:

1. **IT-Driven Personnel Management System**:

   Automate the tracking of personnel training and certification statuses, with reminders for periodic updates and refresher courses.

2. **Comprehensive Onboarding Program**:

   Cover all POCT equipment operations and workflows, ensuring theoretical and practical training for new employees.

3. **Assessment and Authorization Mechanism**:

   Require operators to pass an examination to obtain operational authorization.

3. Patient Identification Verification - Patient Misidentification

Issue Description: Errors in patient identification can lead to incorrect assignment of test results, causing misdiagnosis or inappropriate treatment.

Hospital Situation:

- **Peking University Shenzhen Hospital**: During peak hours, some nursing stations do not strictly follow the patient identification protocol.
- **Wuhan Third Hospital**: Patient identification information is manually recorded, increasing the risk of errors and omissions.

Scoring Analysis:

- **Severity Score (S)**: Critical (4)
  - *Reason*: Misidentification directly results in misdiagnosis or inappropriate treatment, particularly in critical value scenarios.
- **Probability Score (P)**:
  - *Peking University Shenzhen Hospital*: Improbable (2)
    - *Reason*: Patient identification processes are generally followed, with occasional issues during peak hours.
  - *Wuhan Third Hospital*: Improbable (2)
    - *Reason*: Manual recording increases the risk, but no major issues have been reported.

Risk Level:

Peking University Shenzhen Hospital: Acceptable (Improbable, Critical)

Wuhan Third Hospital: Acceptable (Improbable, Critical)

Control Measures:

No further action plan is required at this time.

4. Improper Specimen Handling - Inadequate Protocols for Specimen Management

Issue Description: Improper specimen management (e.g., incorrect labeling, delayed transportation) can result in inaccurate test results or lost specimens.

Hospital Situation:

Peking University Shenzhen Hospital: Some nursing stations have not fully standardized specimen management workflows, with occasional transportation issues.

Wuhan Third Hospital: Specimen management relies on manual handling, with labeling and transportation lacking systematic procedures.

Scoring Analysis:

Severity Score (S): Serious (3)

Reason: Improper specimen handling can cause deviations or delays but is unlikely to directly threaten life.

Probability Score (P):

Peking University Shenzhen Hospital: Occasional (3)

Reason: Processes are generally standardized, but peak-hour issues are relatively common.

Wuhan Third Hospital: Occasional (3)

Reason: Manual processes present a moderate probability of error.

Risk Level:

Peking University Shenzhen Hospital: Acceptable (Occasional, Serious)

Wuhan Third Hospital: Acceptable (Occasional, Serious)

Control Measures:

No further action plan is required at this time.

---

5. Instrument - Equipment Failure or Sensor Malfunction

Issue Description: Equipment failure or sensor malfunction may disrupt testing or lead to biased results.

Hospital Situation:

Peking University Shenzhen Hospital: Some equipment lacks timely maintenance, with occasional failures affecting testing.

Wuhan Third Hospital: Limited equipment availability leads to longer repair cycles in case of failure.

Scoring Analysis:

Severity Score (S): Serious (3)

Reason: Equipment failure can cause result deviations but is unlikely to directly threaten life.

Probability Score (P):

Peking University Shenzhen Hospital: Remote (2)

Reason: Most equipment is well-maintained, with delays affecting only a few units.

Wuhan Third Hospital: Remote (2)

Reason: Failure probability is low, but repair times are prolonged.

Risk Level:

Peking University Shenzhen Hospital: Acceptable (Remote, Serious)

Wuhan Third Hospital: Acceptable (Remote, Serious)

Control Measures:

No further action plan is required at this time.

6. Reagents - Reagent Failure or Batch Variation

Issue Description: Reagent failure or variations between batches can cause inconsistent or inaccurate test results.

Hospital Situation:

Peking University Shenzhen Hospital: Reagent storage conditions do not fully meet standards, with occasional temperature fluctuations or cold-chain interruptions.

Wuhan Third Hospital: Inventory management is inadequate, with frequent mixing of reagent batches and insufficient validation.

Potential Consequences:

Reagent performance fluctuations may result in test deviations or quality control failures, affecting clinical diagnosis.

Scoring Analysis:

Severity Score (S): Serious (3)

Reason: Reagent failures or variations may produce inaccurate results but typically do not directly endanger patient safety.

Probability Score (P):

Peking University Shenzhen Hospital: Remote (2)

Reason: Storage conditions are generally compliant, but occasional temperature issues arise.

Wuhan Third Hospital: Occasional (3)

Reason: Inventory management is less robust, with frequent reagent mixing.

Risk Level:

Peking University Shenzhen Hospital: Acceptable (Remote, Serious)

Wuhan Third Hospital: Acceptable (Occasional, Serious)

Control Measures:

No further action plan is required at this time.

7. Calibration - Insufficient Calibration

Issue Description: Insufficient calibration may degrade equipment performance, resulting in inaccurate test results.

Hospital Situation:

Peking University Shenzhen Hospital: Calibration of some high-frequency equipment is not updated on time, with incomplete calibration records.

Wuhan Third Hospital: Calibration primarily relies on the supplier, and internal calibration procedures are not well-established.

Potential Consequences:

Uncalibrated equipment may lead to systematic errors in test results, especially affecting critical parameter measurements.

Scoring Analysis:

- **Severity Score (S)**: Serious (3)
  - *Reason*: Insufficient calibration affects test accuracy but does not directly lead to critical outcomes.
- **Probability Score (P)**:
  - *Peking University Shenzhen Hospital*: Occasional (3)
    - *Reason*: Some equipment calibration is delayed, but such issues are infrequent.
  - *Wuhan Third Hospital*: Probable (4)
    - *Reason*: Calibration procedures are incomplete, and equipment often remains uncalibrated for extended periods.

Risk Level:

- *Peking University Shenzhen Hospital*: Acceptable (Occasional, Serious)
- *Wuhan Third Hospital*: Unacceptable (Probable, Serious)

Next Steps:

1. **Establish Internal Calibration Procedures**:
   - Develop detailed calibration plans to ensure timely calibration of all equipment.
2. **Strengthen Calibration Record Management**:

Use a digital record system to track and review calibration history.

8. Internal Quality Control (IQC) - Inadequate Quality Control

Issue Description: Inadequate internal quality control (IQC) may result in inconsistent equipment test results, increasing the risk of data deviations.

Hospital Situation:

Peking University Shenzhen Hospital: Some testing sites do not conduct daily IQC or maintain adequate frequency.

Wuhan Third Hospital: Failures in IQC are not addressed promptly, leading to the accumulation of issues.

Scoring Analysis:

Severity Score (S): Serious (3)

Reason: Insufficient IQC can cause systematic errors but does not usually pose immediate threats to patient safety.

Probability Score (P):

Peking University Shenzhen Hospital: Occasional (3)

Wuhan Third Hospital: Probable (4)

Risk Level:

Peking University Shenzhen Hospital: Acceptable (Occasional, Serious)

Wuhan Third Hospital: Unacceptable (Probable, Serious)

Next Steps:

1. **Increase IQC Frequency and Strengthen Oversight**:

   Ensure daily IQC is performed according to standards, with periodic result reviews.

2. **Establish a Mechanism for IQC Failure Response**:

   Implement a rapid response process to address IQC anomalies promptly.

9. External Quality Assessment (EQA) - Failure to Implement EQA

Issue Description: Failure to participate in external quality assessment (EQA) or implement result comparison mechanisms may delay the identification of equipment deviations.

Hospital Situation:

Peking University Shenzhen Hospital: Departments such as endocrinology participate in EQA; however, some devices are excluded and rely solely on internal QC data and periodic comparisons with biochemical analyzers.

Wuhan Third Hospital: EQA has not been implemented in clinical settings, and quality assessments rely on internal QC data and periodic comparisons with laboratory analyzers.

Scoring Analysis:

Severity Score (S): Serious (3)

Reason: Lack of EQA may lead to long-term equipment deviations but does not typically threaten life.

Probability Score (P):

Peking University Shenzhen Hospital: Remote (2)

Wuhan Third Hospital: Remote (2)

Risk Level:

Peking University Shenzhen Hospital: Acceptable (Remote, Serious)

Wuhan Third Hospital: Acceptable (Remote, Serious)

Control Measures:

No further action plan is required at this time.

10. Data Management - Manual Errors, Patient Misidentification, or Data Loss

Issue Description: Data management errors may lead to incorrect result assignments or data loss, affecting diagnosis and treatment.

Hospital Situation:

Peking University Shenzhen Hospital: Some nursing stations still rely on manual data entry, posing higher risks.

Wuhan Third Hospital: Entirely dependent on manual records, increasing the likelihood of errors and data loss.

Scoring Analysis:

Severity Score (S): Serious (3)

Reason: Data errors can impact diagnostic decisions but do not usually pose immediate life-threatening risks.

Probability Score (P):

Peking University Shenzhen Hospital: Probable (4)

Wuhan Third Hospital: Probable (4)

Risk Level:

Peking University Shenzhen Hospital: Unacceptable (Probable, Serious)

Wuhan Third Hospital: Unacceptable (Probable, Serious)

Next Steps:

1. **Introduce Automated Data Management Systems**:

   Implement LIS systems to reduce manual data entry.

2. **Barcode Scanning Technology**:

Ensure patient data and test results are automatically linked using barcodes.

11. Quality Indicators (QIs) - Insufficient Ongoing Review of Quality Indicators

Issue Description: Insufficient monitoring and review of quality indicators (QIs) may result in the accumulation of latent issues, delaying process optimization.

Hospital Situation:

Peking University Shenzhen Hospital: POCT-related data collection and analysis are delayed, relying on retrospective statistical reviews despite laboratory accreditation.

Wuhan Third Hospital: POCT-related QIs are neither systematically defined nor monitored.

Scoring Analysis:

Severity Score (S): Serious (3)

Reason: Lack of QI monitoring may delay process improvements but does not directly affect safety.

Probability Score (P):

Peking University Shenzhen Hospital: Occasional (3)

Wuhan Third Hospital: Occasional (3)

Risk Level:

Peking University Shenzhen Hospital: Acceptable (Occasional, Serious)

Wuhan Third Hospital: Acceptable (Occasional, Serious)

Control Measures:

No further action plan is required at this time.

Post-Analytical Phase Risk Assessment Report

1. Result Reception - Test Results Not Reported to Appropriate Personnel

Issue Description:

Some test results are not delivered promptly to the relevant personnel or patients, particularly during night shifts or peak hours, which may delay diagnosis and treatment.

Hospital Situation:

- **Peking University Shenzhen Hospital**: Some nursing stations fail to deliver reports on time due to complex workflows or staff workload during busy periods.
- **Wuhan Third Hospital**: Result reporting mainly relies on manual recording and delivery, which increases the risk of omission or delay.

Potential Consequences:

Delayed result reporting may reduce diagnostic and treatment efficiency but typically does not compromise patient safety.

Scoring Analysis:

- **Severity Score (S)**: Minor (2)
  - *Reason*: Delays mainly affect efficiency and do not directly threaten patient safety.
- **Probability Score (P)**:
  - *Peking University Shenzhen Hospital*: Remote (2)

Reason: Existing processes are relatively sound, with occasional delays during peak hours.

Wuhan Third Hospital: Remote (2)

Reason: Manual workflows present a risk, but the probability is relatively low.

Risk Level:

Peking University Shenzhen Hospital: Acceptable (Remote, Minor)

Wuhan Third Hospital: Acceptable (Remote, Minor)

Control Measures:

No further action plan is required at this time.

2. Result Interpretation - Inappropriate Interpretation of Results

Issue Description: Improper interpretation of test results may lead to misdiagnosis or treatment delays, particularly for critical values or complex cases.

Hospital Situation:

Peking University Shenzhen Hospital: Some physicians rely on experience for interpretation and do not fully reference device manuals or standardized protocols.

Wuhan Third Hospital: Physicians and operators have insufficient training in result interpretation, with limited ability to analyze abnormal values.

Potential Consequences:

Errors in interpretation may result in misdiagnosis or incorrect treatment, significantly impacting diabetes or emergency patients.

Scoring Analysis:

- **Severity Score (S)**: Serious (3)
  - *Reason*: Misinterpretation affects diagnosis and treatment but rarely results in direct patient harm.
- **Probability Score (P)**:
  - *Peking University Shenzhen Hospital*: Remote (2)
    - *Reason*: Most physicians have sufficient experience, making errors unlikely.
  - *Wuhan Third Hospital*: Remote (2)
    - *Reason*: Training deficiencies are offset by consultation practices, reducing the risk of subjective misinterpretation.

Risk Level:

- *Peking University Shenzhen Hospital*: Acceptable (Remote, Serious)
- *Wuhan Third Hospital*: Acceptable (Remote, Serious)

Control Measures:

No further action plan is required at this time.

3. Interferences - Potential Interferences Affecting Glucose Measurement Accuracy

Issue Description: Test results may be affected by interferences such as hematocrit levels, medications, or other factors, reducing accuracy.

Hospital Situation:

Peking University Shenzhen Hospital: Operator proficiency in recognizing and managing interferences varies.

Wuhan Third Hospital: Clear guidelines for managing interferences are lacking, and operator awareness is limited.

Potential Consequences:

Interferences may lead to biased results or misinterpretation, increasing the risk of misdiagnosis.

Scoring Analysis:

Severity Score (S): Serious (3)

Reason: Unrecognized interferences may lead to incorrect treatment but typically do not result in severe outcomes.

Probability Score (P):

Peking University Shenzhen Hospital: Remote (2)

Wuhan Third Hospital: Remote (2)

Risk Level:

Peking University Shenzhen Hospital: Acceptable (Remote, Serious)

Wuhan Third Hospital: Acceptable (Remote, Serious)

Control Measures:

No further action plan is required at this time.

4. Result Reporting - Incomplete Elements in POCT Reports

Issue Description: Some POCT reports lack essential information (e.g., patient information, test time, operator information), which may affect result traceability and interpretation.

Hospital Situation:

Peking University Shenzhen Hospital: Some reports omit operator information or test type.

Wuhan Third Hospital: Manual recording often results in omissions, impacting report completeness.

Scoring Analysis:

Severity Score (S): Serious (3)

Reason: Missing information can make result traceability challenging but does not directly affect patient safety.

Probability Score (P):

Peking University Shenzhen Hospital: Occasional (3)

Wuhan Third Hospital: Occasional (3)

Risk Level:

Peking University Shenzhen Hospital: Acceptable (Occasional, Serious)

Wuhan Third Hospital: Acceptable (Occasional, Serious)

Control Measures:

No further action plan is required at this time.

5. Critical Value Reporting - Delayed or Incorrect Reporting of Critical Values

Issue Description: Delayed or incorrect reporting of critical values may result in treatment delays or inappropriate medical decisions.

Hospital Situation:

Peking University Shenzhen Hospital: Critical value management processes are well-established, with occasional delays during night shifts or peak periods.

Wuhan Third Hospital: Critical value reporting processes are incomplete, with risks of omissions or delays.

Scoring Analysis:

Severity Score (S): Catastrophic (5)

Reason: Delayed critical values can directly threaten patient life.

Probability Score (P):

Peking University Shenzhen Hospital: Improbable (1)

Wuhan Third Hospital: Improbable (1)

Risk Level:

Peking University Shenzhen Hospital: Acceptable (Improbable, Catastrophic)

Wuhan Third Hospital: Acceptable (Improbable, Catastrophic)

Control Measures:

No further action plan is required at this time.

6. Biosafety and Infection Control - Failure to Implement Reasonable Protective Measures

Issue Description: Inadequate biosafety and infection control measures may expose medical staff or patients to infection risks.

Hospital Situation:

Peking University Shenzhen Hospital: Some nursing stations do not strictly follow hand hygiene or equipment cleaning protocols.

Wuhan Third Hospital: Infection control measures are incomplete, with insufficient enforcement in high-risk areas.

Scoring Analysis:

Severity Score (S): Serious (3)

Reason: Inadequate infection control may cause cross-infection but does not typically threaten life directly.

Probability Score (P):

Peking University Shenzhen Hospital: Occasional (3)

Wuhan Third Hospital: Occasional (3)

Risk Level:

Peking University Shenzhen Hospital: Acceptable (Occasional, Serious)

Wuhan Third Hospital: Acceptable (Occasional, Serious)

Control Measures:

No further action plan is required at this time.
