# Supplementary material for: Risk management in POCT blood glucose monitoring: FMEA approach aligned with ISO 15189:2022
Source: PLoS One. 2025 Mar 10;20(3):e0319817. doi: 10.1371/journal.pone.0319817 (PMC11892846; doi:10.1371/journal.pone.0319817)
Supplement: Attachment — Submitted filename: Point by point responses to the reviewers(1).doc [file pone.0319817.s003.doc]

### ****1. Is the manuscript technically sound, and do the data support the conclusions?****

**Reviewer #1: Yes**
**Reviewer #2: No**

**Response**:
Thank you for your comments. We acknowledge Reviewer #2's concern regarding the sufficiency of data to support the conclusions. The study utilizes the **Failure Mode and Effects Analysis (FMEA)** approach, which primarily relies on the clinical expertise, hands-on experience, and consensus-based brainstorming of a multidisciplinary team to identify and assess risks. This methodology inherently focuses on qualitative risk identification rather than extensive quantitative data collection, which is a recognized limitation of the FMEA approach itself. We have provided relevant information on the general process of implementing the FMEA method in the Supporting Information (S2 File).

To address this, we emphasize that our multidisciplinary team consisted of members from different hospitals, including frontline clinical physicians, nursing staff, laboratory technicians, and POCT managers. Their diverse professional backgrounds and extensive practical experience in POCT management provided robust expertise that partially compensates for the lack of detailed quantitative data.

In addition, we have revised the **Discussion** section to explicitly acknowledge the inherent limitations of the FMEA approach, including its reliance on expert judgment and the absence of rigorous quantitative data, and discuss the potential impact of this limitation on our conclusions. Furthermore, we will include suggestions for future research to incorporate more quantitative methodologies alongside FMEA to enhance the robustness of the findings (lines 258-268).

### ****2. Has the statistical analysis been performed appropriately and rigorously?****

**Reviewer #1: Yes**
**Reviewer #2: I Don’t Know**

**Response**:
We appreciate Reviewer #2's comment and agree that the manuscript could provide more clarity on the use of statistical analysis. As the study adopts the FMEA approach, the evaluation of risks is based on qualitative assessments rather than statistical analyses, as discussed in the Discussion section (lines 258-268). Risk levels are determined by expert consensus using a risk matrix that incorporates the probability and severity of potential failures, as explained in the Table 1 (line 142).

**3. Have the authors made all data underlying the findings in their manuscript fully available?**

**Reviewer #1: Yes**
**Reviewer #2: No**

**Response**:
We thank Reviewer #2 for raising this concern. The nature of FMEA as a qualitative assessment method means that the findings are derived from expert evaluations and consensus rather than raw numerical data. However, we acknowledge the importance of transparency in reporting. To address this issue, we have included all relevant materials as Supporting information in the revised manuscript. Specifically, S1 File provides a detailed discussion of the risk matrix, while S2 File includes documentation of the brainstorming session outcomes and the expert consensus process.

### ****4. Is the manuscript presented in an intelligible fashion and written in standard English?****

**Reviewer #1: Yes**
**Reviewer #2: Yes**

**Response**:
Thank you for the positive feedback regarding the clarity and quality of the manuscript's language. We will carefully proofread the revised version to correct any typographical or grammatical errors and ensure that the language remains clear and unambiguous.

### ****5. General Comments to the Author****

**Response**:
We greatly appreciate the reviewers' constructive feedback. The following key revisions will be made to address the concerns raised:

1. **Acknowledging Methodological Limitations**: The limitations of the FMEA approach, including its reliance on qualitative data and expert consensus, will be explicitly discussed in the manuscript.
2. **Enhancing Data Transparency**: Supplementary materials, including detailed risk matrices and expert consensus documentation, will be added to improve the transparency and replicability of the study.
3. **Clarifying the Use of Statistical Analysis**: The manuscript will explicitly state that statistical analyses were not employed due to the qualitative nature of the FMEA approach, and the implications of this choice will be discussed.
4. **Language and Presentation**: A thorough review of the manuscript will be conducted to ensure it meets the highest standards of clarity and readability.

### ****Reviewer #1: The use of POCT systems in laboratory medicine is increasingly widespread. Having correct and rapid laboratory data brings a great advantage to the clinician. The manuscript analyzes the critical issues of these systems and provides useful information. It's a good article.****

**Response:**
Thank you for your positive feedback regarding the significance of POCT systems and the value of our manuscript. We appreciate your recognition of the importance of our work in addressing critical issues related to POCT systems. Your encouraging comments reinforce the value of our findings and their potential to contribute to the clinical application of POCT systems.

### ****Reviewer #2: General: Well written. Specific comments:****

#### ****1. line 29: "in clinical settings" – however, the study was not conducted in a clinical setting, but in a clinical laboratory setting (line 111). This can only simulate a “real” clinical setting. The study does not reflect “real life” POCT. This should be clarified and discussed.****

**Response:**
Thank you for highlighting this important distinction. We agree that the study was conducted in a clinical laboratory setting, which only simulates a "real-life" clinical environment. The text has been revised to specify that the study was conducted in a “clinical laboratory setting” rather than a general “clinical setting” (lines 29,71,105,132). We have elaborated on this in the discussion section (lines 262-265).

#### ****2. line 141/Table 1: Probability – the differences should be described. Are the probabilities based on actual frequencies (e.g., 10^-6) or purely descriptive? Severity: The description of the five different severity levels should be included – e.g., Catastrophic: Does this mean “Death or life-threatening injury or illness”?****

**Response:**
Thank you for raising these important points regarding the probability and severity levels in our risk matrix. We appreciate the opportunity to clarify and refine these aspects to improve the transparency and applicability of our findings.

**Probability Levels:**
We acknowledge that the probabilities used in this study were descriptive estimates rather than being derived from actual observed frequencies. These estimates were determined through expert consensus during FMEA brainstorming sessions, reflecting practical constraints in performing real-time risk evaluations in clinical practice.

To enhance clarity, we have updated the manuscript to include the following refined probability definitions based on real-world frequency classifications:

1. **Frequent**: Occurs once per week or more frequently.
2. **Probable**: Occurs once per month.
3. **Occasional**: Occurs once per quarter.
4. **Remote**: Occurs once per year.
5. **Improbable**: Occurs once every 5 years or more.

**Severity Levels:**
Thank you for pointing out the need for more detailed descriptions of the severity levels in Table 1. After review, we agree that the previous definitions could be refined to better align with the clinical implications of POCT blood glucose monitoring. Following consultations with relevant literature and ISO 15189:2022 standards, we have revised the severity levels as follows:

1. **Negligible**: No impact on patient safety or clinical decisions.
2. **Minor**: Minimal impact requiring repeat testing or minor delays, no harm to patient.
3. **Serious**: Moderate impact causing diagnostic delays or inaccuracies, not life-threatening.
4. **Critical**: Significant impact leading to diagnostic errors or inappropriate treatment, potential patient harm.
5. **Catastrophic**: Severe impact, such as missed critical condition diagnosis, resulting in permanent harm or death.

These definitions have been added to the manuscript under the **Table 1**(line 142), see S1 for details.

#### ****3. line 154-159: “Inadequate” and “insufficient” appear several times. What is the difference?****

**Response:**

Thank you for pointing out the potential inconsistency in the use of the terms “inadequate” and “insufficient” personnel training. We appreciate this observation and agree that consistent terminology is essential for clarity.

In the revised manuscript, we have standardized the use of these terms based on the following definitions: In the effectiveness assessment, inadequate refers to conditions where the standard is entirely unmet, while insufficient refers to conditions where partial compliance exists but improvements are necessary (lines 157-159). In addition, we have revised the relevant section to consistently use the term “insufficient personnel training” for both Peking University Shenzhen Hospital and Wuhan Third Hospital.

#### ****4. line 161/Table 2: Are the described probabilities based on actual observations/data – or estimates?****

#### **Response:**

Thank you for raising this important point. We confirm that the probabilities described in Table 2 are **expert-derived estimates,** rather than being based on actual observed frequency data. These estimates were based on the collective clinical experience of the team and observations from real-world clinical practice. The text has been updated in discussion section (lines 204-205).

**5. line 161/Table 2: You mention “validation” on several occasions. Do you mean “verification”?**

**Response:**

Thank you for this valuable observation. Upon review, we have updated the manuscript by replacing “performance validation” with “performance verification” in accordance with ISO 15189:2022 standards terminology (lines 38,164,180,202,209,211,241,278; Table 2, Table 3).

**6. line 161/Table 2/Analytical-Data Management: The implemented risk control measure is “Patient information and test results are entered manually and verified.” Why not use scanning of barcodes and integration with LIS?**

**Response:**

#### Thank you for this insightful observation. We agree that the implementation of barcode scanning and LIS integration is a highly effective measure to reduce human errors in data management. However, in this study, due to limitations in resources and infrastructure availability at Wuhan Third Hospital, manual verification remains the current practice.

#### We have also clarified this point in the Discussion section:

#### Due to limitations in resources and infrastructure availability at Wuhan Third Hospital, manual verification remains the current practice (lines 219-221).

#### ****7. line 181/Table 3: Insufficient personnel training – “IT driven” for Peking and “Manual tracking” for Wuhan. Why different?****

**Response:**

Thank you for highlighting this point. The difference between “IT-driven” solutions at Peking University Shenzhen Hospital and “Manual tracking” at Wuhan Third Hospital arises primarily from disparities in resource availability and infrastructure between the two hospitals.

**Peking University Shenzhen Hospital**: This hospital has access to IT-driven solutions, such as automated tracking systems for personnel training and certification, which enable efficient monitoring and reminders for training updates.

**Wuhan Third Hospital**: Due to resource constraints, personnel training and certification are tracked manually, relying on logbooks and periodic audits.

**We have clarified these differences in the Table 3: Insufficient personnel training. And we have added** in the Discussion section: As with the measures in data management, IT-driven solutions in personnel training can enhance the efficiency and accuracy of training management, ultimately improving the quality of POCT services. However, in resource-limited environments, the following measures remain essential to mitigate risks: manual tracking of personnel, enhanced supervision with mentorship, and ongoing performance assessments (lines 232-237).
.

**8. line 181/Table 3: Data management – “Automated” for Peking and “Manual verification” for Wuhan. Why different?**

**Response:**

Thank you for raising this important point. The difference between “Automated” data management at Peking University Shenzhen Hospital and “Manual verification” at Wuhan Third Hospital reflects disparities in infrastructure and resource availability between the two hospitals.

**Peking University Shenzhen Hospital**: Automated systems are used for data management, enabling seamless integration with laboratory information systems (LIS). These systems ensure accurate and efficient data entry, storage, and retrieval while minimizing human error.

**Wuhan Third Hospital**: Due to resource constraints, data management primarily relies on manual verification processes. Patient information and test results are manually entered and cross-checked by staff, which is labor-intensive and prone to occasional errors.

To address this, we have clarified the following in the revised manuscript:

Due to limitations in resources and infrastructure availability at Wuhan Third Hospital, manual verification remains the current practice. To mitigate actual risks in resource-limited environments like Wuhan Third Hospital, the risk management team recommends maintaining manual verification, regular manual audits, and paper-based backups. These measures aim to ensure the accuracy and integrity of data management processes in the absence of fully implemented IT systems (lines 219-225).

**9. line 193: “…but also enhanced the overall reliability of POCT….”: How do you support this claim? Data? Subjective evaluation?**

**Response:**

Thank you for raising this concern. The statement regarding the improvement in the overall reliability of POCT was based on **subjective evaluations** provided by the multidisciplinary team during the follow-up period. These evaluations reflected the team’s observations and feedback on the impact of the implemented control measures.

We have added an explanation of this in the Discussion section:

Due to limitations in resources and infrastructure availability at Wuhan Third Hospital, manual verification remains the current practice. To mitigate actual risks in resource-limited environments like Wuhan Third Hospital, the risk management team recommends maintaining manual verification, regular manual audits, and paper-based backups. These measures aim to ensure the accuracy and integrity of data management processes in the absence of fully implemented IT systems (lines 219-225).

**10. line 200-206: If manual data entry was a risk at Peking, why was it not considered a risk at Wuhan?**

**Response:**

Thank you for raising this question. Manual data entry is indeed a potential risk at both hospitals. Refer to **Line 172/Table 2 - Analytical: Data Management** for further details.

However, in this study, it was prioritized at Peking University Shenzhen Hospital due to its higher workload and previous reliance on manual processes. The differences in infrastructure and resource availability between the two hospitals have led to variations in the improvement measures implemented. The relevant measures have been addressed in detail in the response to Question 8.

The text has been updated in the discussion section:

Data management issues posed significant risks at Peking University Shenzhen Hospital due to its higher workload and previous reliance on manual processes (lines 214-215).

**11. line 243-251: “…highlights the efficiency of FMEA…” and “…have significantly improved….”: Where is the data to support these claims?**

**Response:**

Thank you for pointing out the need for empirical support to substantiate the claims regarding the efficiency of FMEA and the observed improvements. We acknowledge that “significantly” is a strong term that requires quantitative data for validation. To address this, the term “significantly” has been replaced with “noticeably” **in the Results Section** to better reflect the qualitative improvements observed during the study without implying statistical validation (line 279).
